# Design and Implementation of Autonomous and Non-Autonomous Time-Delay Chaotic System Based on Field Programmable Analog Array

**DOI:** 10.3390/e21050437

**Published:** 2019-04-26

**Authors:** Han-Ping Hu, Xiao-Hui Liu, Fei-Long Xie

**Affiliations:** 1 School of Artificial Intelligence and Automation, Huazhong University of Science and Technology, 1037 Luoyu Road, Wuhan 430074, China; 2 Key Laboratory of Image Information Processing and Intelligent Control, Ministry of Education, Wuhan 430074, China

**Keywords:** chaos, time-delay, chaotic secure communications, FPAA, Ikeda system, Duffing system

## Abstract

Time-delay chaotic systems can have hyperchaotic attractors with large numbers of positive Lyapunov exponents, and can generate highly stochastic and unpredictable time series with simple structures, which is very suitable as a secured chaotic source in chaotic secure communications. But time-delay chaotic systems are generally designed and implemented by using analog circuit design techniques. Analog implementations require a variety of electronic components and can be difficult and time consuming. At this stage, we can now solve this question by using FPAA (Field-Programmable Analog Array). FPAA is a programmable device for implementing multiple analog functions via dynamic reconfiguration. In this paper, we will introduce two FPAA-based design examples: An autonomous Ikeda system and a non-autonomous Duffing system, to show how a FPAA device is used to design programmable analog time-delay chaotic systems and analyze Shannon entropy and Lyapunov exponents of time series output by circuit and simulation systems.

## 1. Introduction

In security communication, the cryptogram generator is a key device. It is showed that in chaotic security communications, this cryptogram generator can be a chaotic system. In chaotic security communications, the utility of the chaotic system is “encryption”, thus it is valuable to construct a proper chaotic system for chaotic security communication. A time delayed chaotic system has a simple structure and a hyperchaotic attractor in phase space, which provides a higher level of security in chaotic secure communications [1,2,3,4]. Therefore the design and implementation of a time-delayed chaotic system is of high practical importance for increasing the safety of secure communication.

FPAA (Field Programmable Analog Array) is a dynamically programmable Analog Signal Processor device. It has integrator, comparator, amplifier, inverter, multiplier, delay and other blocks. These blocks are constructed from a combination of conventional and switched capacitor circuit elements and are programmed by a host processor [5]. We can easily and flexibly use FPAA’s programming software to design an analog circuit including time-delay chaotic system through the combination of blocks. Then these analog circuits pre-constructed can be realized by being downloaded to the FPAA development board in real time. Therefore, this programmable device is easier, more efficient and more economical than using individual operational amplifiers, resistors, capacitances, analog multipliers and other discrete components used for implementing analog circuit systems.

FPAA has become more and more popular recently [6,7,8,9]. Caponetto used FPAAs to design and implement a fully programmable Chua’s circuit and highlight several advantages of the approach: The design and implementation phases are very simple, and the circuit is totally programmable [10]. Further, Recai Kilic realized FPAA-based Chua’s circuit models and jerk circuit using different nonlinear functions in a programmable and reconfigurable form [11,12]. What’s more, Recai Kilic introduced a universal approach to design and implement programmable analog non-time-delay chaotic systems based on FPAA [13]. After that, Fatma Yildirim Dalkiran and J. C. Sprott realized a fourth-order hyperjerk system based on FPAA [14]. Chunbiao Li designed and implemented chaotic systems with complete amplitude control and constructed infinitely many attractors in a programmable chaotic circuit based on FPAA [15,16]. But the above researchers had not designed and implemented time-delay chaotic systems based on the FPAA. Further, time-delayed chaotic systems can provide a higher level of security in chaotic secure communications compared with non-time-delay chaotic systems, so the design and implementation of a time-delayed chaotic circuit based on FPAA can be very helpful for researchers of chaos secure communication.

Therefore, in this paper, we aim to introduce a universal approach to design and implement programmable analog time-delay chaotic systems based on FPAA. In this context, firstly the design procedure of a FPAA device will be given and then FPAA-based design examples including autonomous and non-autonomous time-delay chaotic circuit models will be introduced. At the same time, we further analyze Shannon entropy and Lyapunov exponents of time series outputs by circuit and simulation systems. We hope that these design notes will be a useful practical guide for researchers who wish to experimentally study time-delay chaotic systems.

## 2. FPAA-Based System Designs

**Figure 1:**
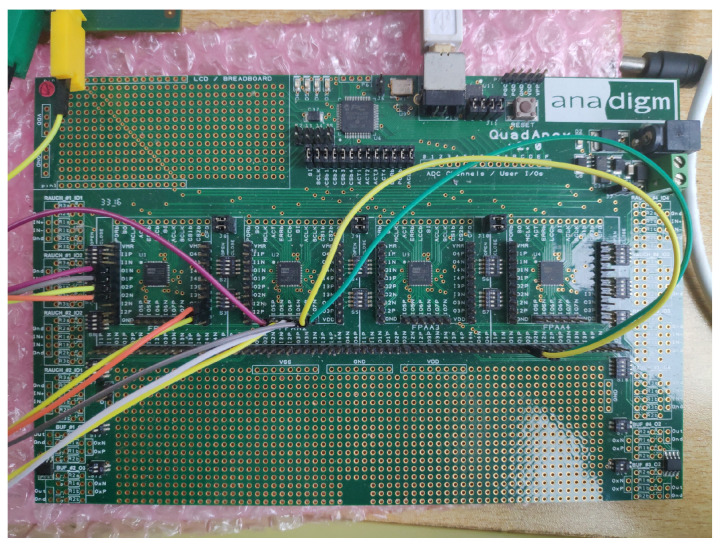
Anadigm QuadApex development board.

Here in this paper we use the newest integrated circuit technique of FPAA to realize time-delay chaotic systems. A FPAA development software named AnadigmDesigner2 was used to design time-delay systems in Windows. An Anadigm QuadApex development board shown in Figure 1 with four AN231E04 chips was used to construct a circuit implementation of time-delay chaotic system.

**Figure 2:**
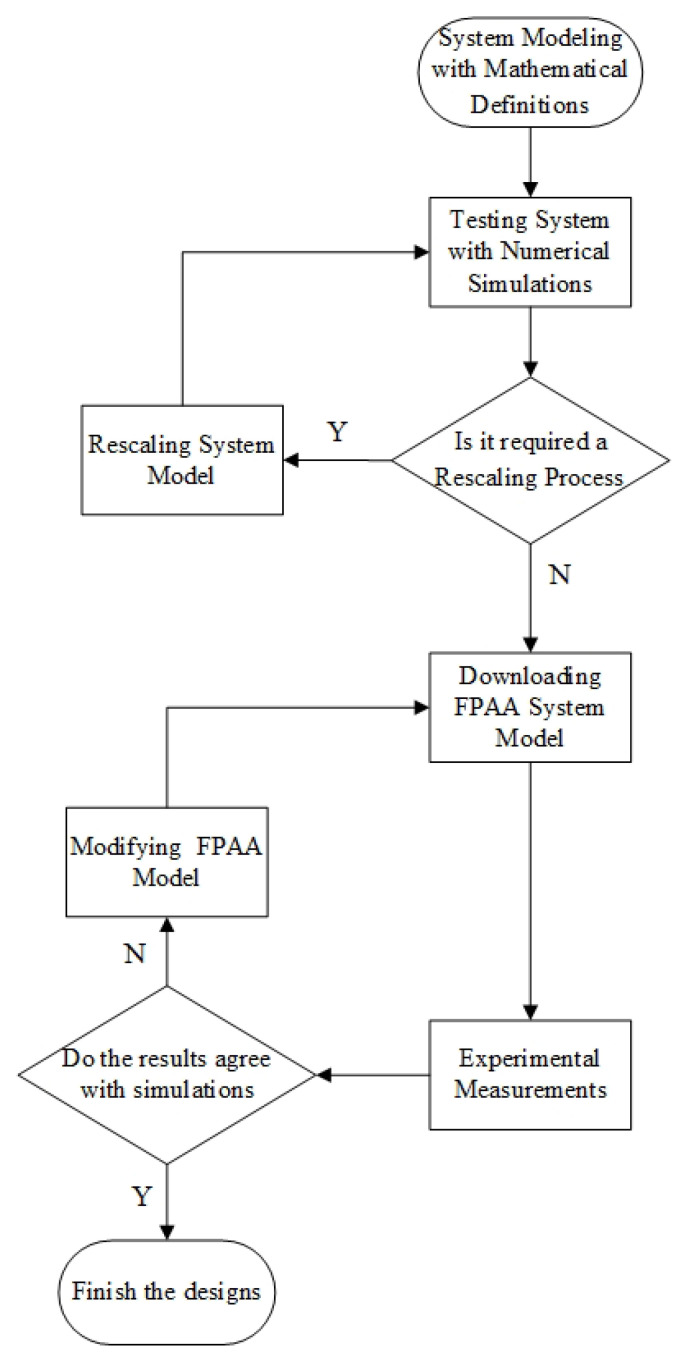
Field-Programmable Analog Array (FPAA)-based design and implementation procedure.

Figure 2 shows the basic flow chart of the FPAA designs. The analog signals of FPAA are limited to the range −3 V to +3 V. Therefore, we test the system with the numerical simulation before using the FPAA design system. Then we decide to rescale the system or not according to the simulation results. If there is something noticeable, we usually use matlab to simulate the system because the simulation tool of FPAA is too slow and the simulation time is too short. Then the system is designed in the FPAA design software, which is similar to the Simulink module of matlab. After setting up the circuit, the configuration information will be downloaded to the FPAA development board by clicking the download button in the software. The experimental results are compared with the simulation results, and if the results are satisfactory, the implementation is finished, otherwise FPAA modeling needs to be modified.

## 3. FPAA-Based Time-Delay Chaotic System Realizations

### 3.1. Autonomous Time-Delay Ikeda System

**Figure 3:**
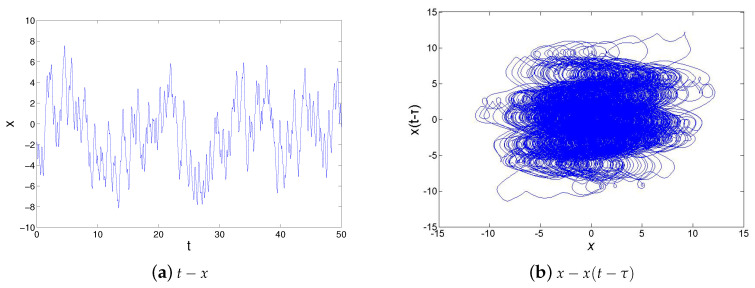
Simulation results of Ikeda system model (1).

Ikeda system is a one-order autonomous time-delay chaotic system [17]. It describes the phase shift in nonlinear optics, and presents a variety of periodic bifurcation and chaotic behaviors. The Ikeda system is one of the few delayed chaotic systems that have been studied deeply. Ikeda’s system is defined by the following state equation:

(1) $$\dot{x}=-\alpha x+\beta \sin(x(t-\tau )),$$

where *x* denote the state variables of the system, α and β are system parameters and τ represents the delay time which plays an important role in the system’s chaos mechanism. These parameters are determined as τ=2,α=1,β=2. Before autonomous Ikeda system implementing, the system defined by Equation (1) is tested with a numerical simulation tool. The numerical simulation results of Ikeda system are illustrated in Figure 3 by adopting the Fourth-Order Runge-Kutta Method in matlab. The maximum value of |x| was more than 3V according to simulation results, System (1) is rescaled to x→10x as following:

(2) $$\dot{x}=-\alpha x+0.1\beta \sin(10x(t-\tau )).$$

**Figure 4:**
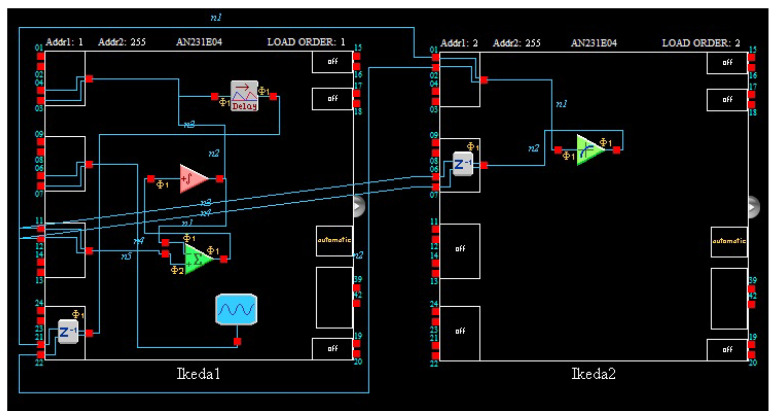
Circuit implementation of the Ikeda system.

According to the Equation (2), the circuit is constructed. sin10x is implemented by a programmable transfer function module. We can design and realize all kinds of nonlinear functions by using the transfer function module. Compared with analog circuit design techniques, it is very easy, efficient and economical to help us realize all kinds of nonlinear systems. At the same time, the delay of circuit was designed by the delay module, so we can change the delay parameter easily by programming. Besides, attention should be paid to the time scale conversion when the analog circuit is constructed by the Equation (2). The delay time is 2 and the integral constant of integrator of FPAA is usually determined as 0.0025 1/us. Therefore, the parameters of the actual delay module are 800 us. The circuit diagram constructed by AD2 software is illustrated in Figure 4.

**Figure 5:**
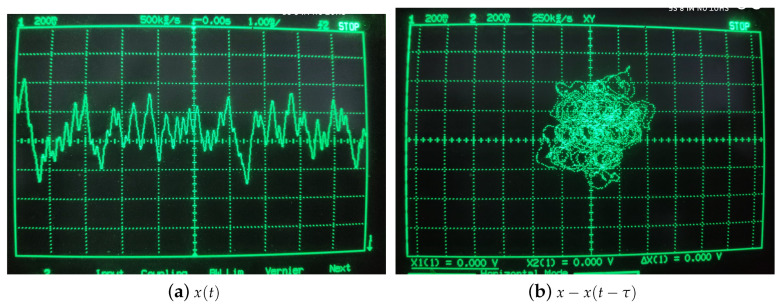
Chaotic signals and phase portrait of the Ikeda system.

**Figure 6:**
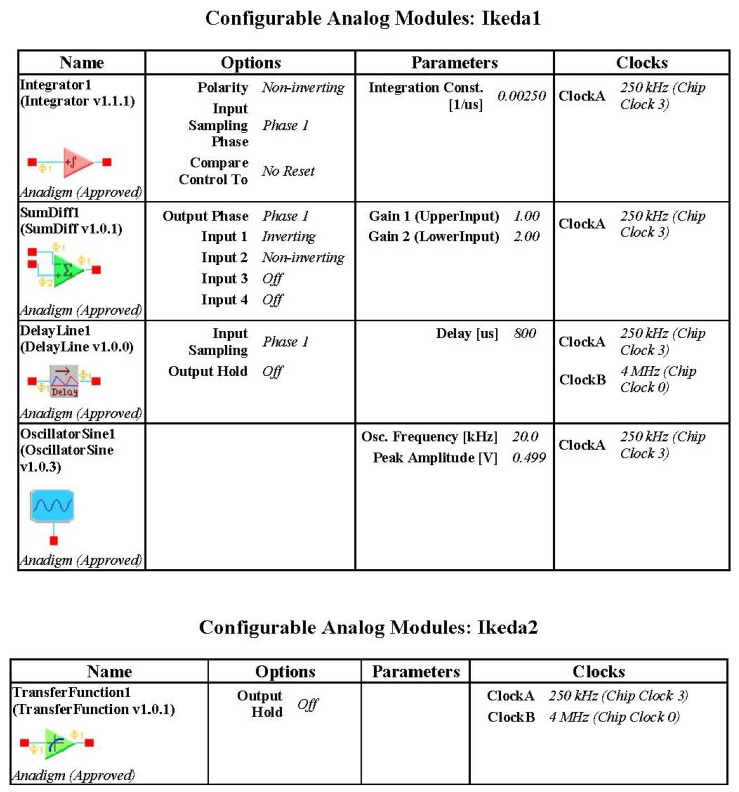
CAM(Configurable Analog Modules) parameters for Ikeda system circuit.

Because the chip resources of FPAA are limited, a chip cannot contain both transfer function module and delay module, it needs two FPAA chips to implement, system state-variable *x* is output by IO1. The chaotic dynamics and the chaotic attractor have been showed in Figure 5. The parameters are showed in Figure 6.

Then we further analyze Shannon entropy and Lyapunov exponents of time series output by Ikeda circuit and simulation system. We sample 130,000 points to be used to test for the Ikeda circuit with a sample rate of 256 kHz, then, the number of point of Ikeda simulation system is also 130,000 and the step of simulation system is 0.01. The results are listed in the Table 1.

Experimental results showed that Shannon entropy and Lyapunov exponents of Ikeda circuits and simulation system are approximately equal. Therefore an autonomous Ikeda chaotic system has been implemented successfully by using FPAA programmable device according to pictorial results and quantitative results.

### 3.2. Non-Autonomous Duffing System

Apart from the autonomous time-delay chaos system implemented by FPAA in the above section, FPAAs are also fit to implement non-autonomous time-delay chaos systems by programming and reconfiguring. In this section, we will introduce how a non-autonomous time-delay chaotic system can be implemented by using a FPAA device.

The Duffing system is one of the most typical and important objects in nonlinear dynamics, because it can model the large deformation or similar properties in an engineering structure. A lot of engineering systems could be described by Duffing or Duffing-based oscillators to enlighten their complicated dynamical behaviors and mechanisms. The time-delay equation of Duffing system is as follows according to this paper [18].

(3) $$\ddot{x}+c\dot{x}-kx+\alpha x^{3}=f\cos\left(\omega t\right)+\mu x\left(t-\tau \right).$$

**Figure 7:**
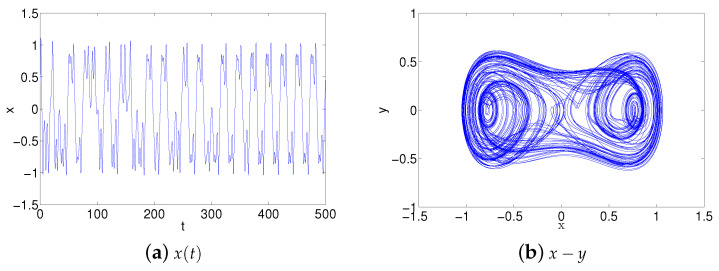
Simulation results of Duffing system model (3) and $y=\dot{x}$.

In the above equation, *x* denote the state variables of the system, α, *c* and *k* are system parameters and τ represents delay time. These parameters are determined as k=1,c=0.2,α=0.5,ω=1.2, τ=0.5,μ=0.05,f=0.5. This system is appropriate for programmable and reconfigurable design and implementation. The new designs are implemented more easily and inexpensively by changing system parameters of the Duffing system flexibly through software. FPAA device has an internal wave generator, it can generate many waves such as sin-waves and square waves, and the frequency, amplitude and other parameters of waves can be changed easily by programming. Therefore, it is non-essential to use an external AC source in FPAA implementation of the Duffing system and the amplitude and frequency parameters of the sin-wave can be easily adjusted by programming. As in the above Ikeda system, the necessary system simulation is required before FPAA modeling. The chaotic dynamics and the chaotic attractor of the simulation have been showed in Figure 7. According to the simulation results the System (3) is rescaled to x→2x as follows:

(4) $$\ddot{x}+c\dot{x}-kx+4\alpha x^{3}=0.5f\cos\left(\omega t\right)+\mu x\left(t-\tau \right).$$

**Figure 8:**
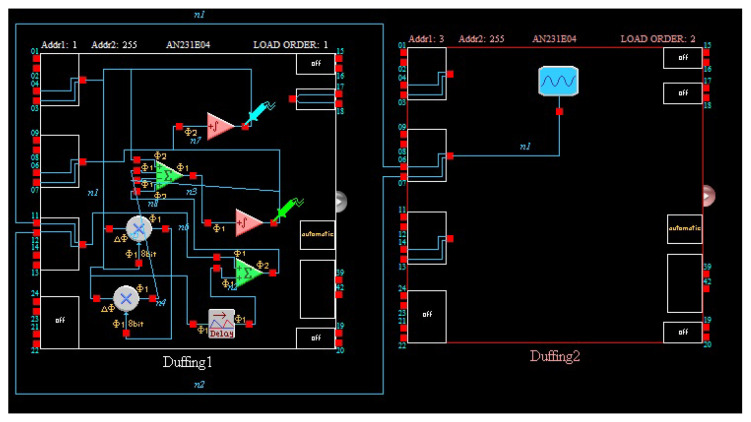
Circuit implementation of Duffing system.

**Figure 9:**
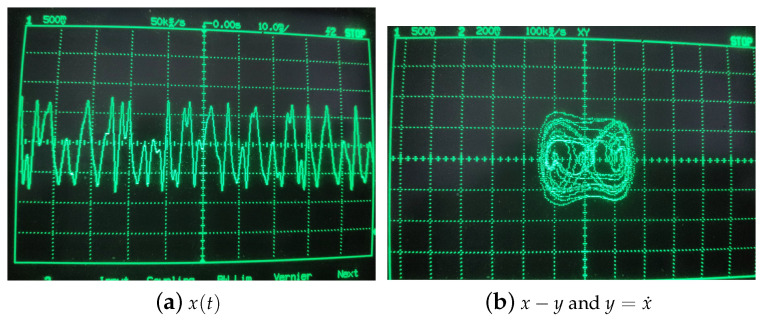
Chaotic signals and phase portrait of Duffing system.

**Figure 10:**
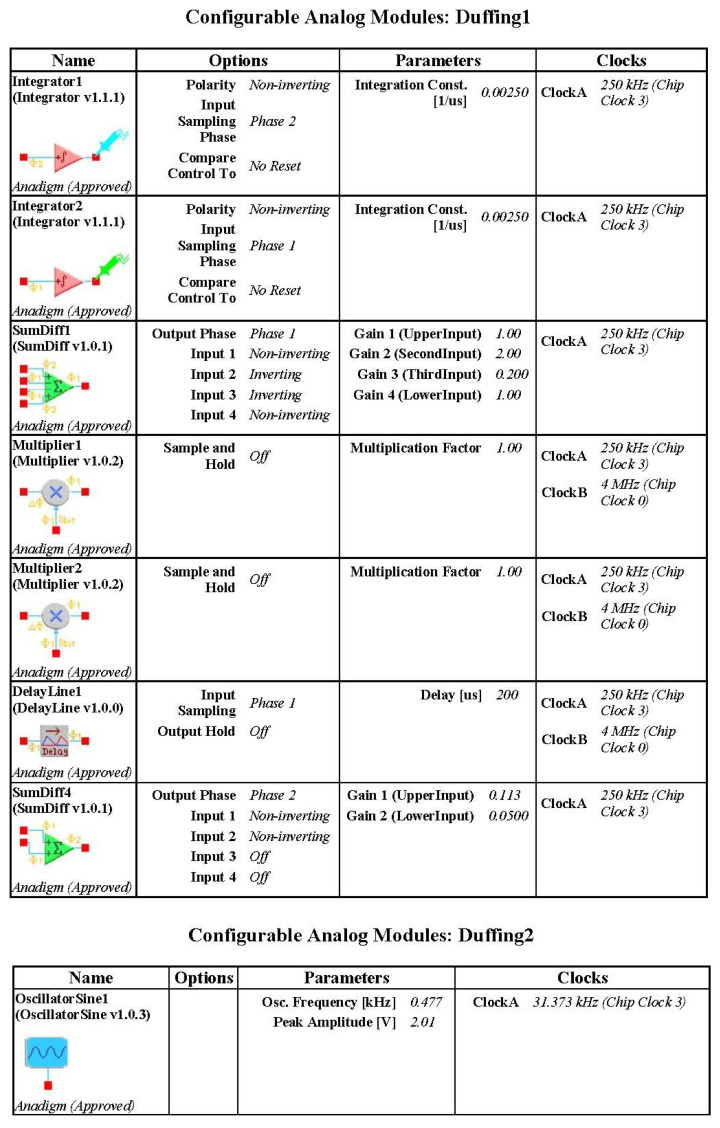
CAM parameters for Duffing system circuit.

Then the Duffing system is modeled with an FPAA software tool. The Duffing system also needs an AC source module, which is different from autonomous time-delay chaos system. Attention should be paid to the time scale conversion when the analog circuit is constructed by the Equation (4). The ω is 1.2 and the integral constant of integrator of FPAA is usually determined as 0.0025 1/us. Therefore, the frequency of the AC source block is 0.477 kHz. This model is downloaded to the FPAA development board via a serial port, and experimental measurements obtained from I/O connections of the FPAA board are illustrated in Figure 8. The results of experiment have been showed in Figure 9 and the circuit parameters are desplayed in Figure 10. After that, we further analyze Shannon entropy and Lyapunov exponents of the time series output by a Duffing circuit and simulation system. Like the Ikeda system, we sample 130,000 points to be used to test for Duffing circuit and the sample rate is 256 kHz. The number of point of Duffing simulation system is 130,000 and the step of simulation system is 0.01. The results are listed in the Table 2.

Experimental results showed that Shannon entropy and Lyapunov exponent of Duffing circuit and simulation system are approximately equal. Therefore non-autonomous duffing chaotic system has also been implemented successfully by using FPAA programmable device according to pictorial results and quantitative results.

## 4. Discussion

Here, we have designed and realized an autonomous time delay Ikeda circuit and non-autonomous time delay Duffing circuit successfully according to the analysis of phase picture, Shannon entropy and Lyapunov exponents. We download time delay chaos circuits pre-designed in software to the development board. After this, different chaos circuits can be implemented in the same development, which greatly reduces our design time. At the same time, the range of realizable chaos circuits based on FPAA is enlarged by constructing chaotic systems with time delay in this paper. This is conducive to researchers of chaos secure communication, because the time-delay chaotic source in chaotic secure communication can be realized by programming, and more importantly, the parameters of chaotic source can be changed easily by a programmable way. Although there are many advantages of FPAA in this paper, there are also limitations, at first, as some complex time-delay chaotic circuits may not be realized because the chip resources of FPAA are limited. Besides, the parameter range of the FPAA delay module is limited, and some time delay chaotic systems with large delay may not be realized. At last, the parameters of the delay used in the time-delay result in discrete delay. Therefore, the time-delay chaotic systems based on FPAA are finite dimension systems.

## 5. Conclusions

In this paper, we chose Ikeda and Duffing models as autonomous and non-autonomous design examples to introduce a universal programmable time-delay chaos system based on FPAA. Experimental results agree with the results obtained from simulation. It shows that this programmable design approach will be very useful in many applications based on time-delay chaotic systems. Many time-delay chaotic systems based on mathematical modeling will not need complex electronic hardware, and the design and implementation of time-delay chaotic systems will be more efficient, simpler and more economical. We hope that these design notes will be useful for researchers who wish to experimentally study time-delay chaotic systems.

## Figures and Tables

**Table 1:** Shannon entropy and Lyapunov exponents of time series output by Ikeda circuit and simulation system.

System	Ikeda Circuit	Ikeda Simulation System
Shannon entropy	5.0632	5.0705
Lyapunov exponents	3.7292	3.9201

**Table 2:** Shannon entropy and Lyapunov exponents of time series output by Duffing circuit and simulation system.

System	Duffing Circuit	Duffing Simulation System
Shannon entropy	5.4782	5.4532
Lyapunov exponents	1.5598	1.9294
